# Dietary Hydroxyproline + Vitamin C and *Eucommia ulmoides* Improved the Growth and Flesh Quality of Pacific White Shrimp (*Litopenaeus vannamei*)

**DOI:** 10.3390/ani15020159

**Published:** 2025-01-09

**Authors:** Xinyi Fei, Xiaoqin Li, Tianyu Huang, Beiping Tan, Xiangjun Leng

**Affiliations:** 1 National Demonstration Centre for Experimental Fisheries Science Education, Shanghai Ocean University, Shanghai 201306, China; 18014925953@163.com (X.F.); xqli@shou.edu.cn (X.L.); 18279139870@163.com (T.H.); 2 Centre for Research on Environmental Ecology and Fish Nutrition (CREEFN), Ministry of Agriculture and Rural Affairs, Shanghai Ocean University, Shanghai 201306, China; 3 Laboratory of Aquatic Nutrition and Feed, College of Fisheries, Guangdong Ocean University, Zhanjiang 524088, China; bptan@126.com

**Keywords:** *Eucommia ulmoides*, white shrimp, vitamin C, *hydroxyproline*, growth performance, flesh quality

## Abstract

The present study aimed to evaluate the dietary effects of hydroxyproline (Hyp) + vitamin C (VC) and *Eucommia ulmoides* (EU) on the growth performance and flesh quality of Pacific white shrimp (*Litopenaeus vannamei*). Based on a practical diet with 200 g/kg fish meal inclusion (control diet), 14 g/kg coated Hyp (50%) + 2 g/kg L-ascorbyl-2-phosphate (38%) (Hyp + VC) or 20 g/kg EU was added to form three iso-nitrogenous diets. Pacific white shrimp with an initial body weight of 0.70 ± 0.10 g were fed the above three diets for 8 weeks. Compared with the control group, the weight gain of shrimp fed with the Hyp + VC diet and the EU diet was increased by 13.9% and 18.4% (*p* < 0.05), while the feed conversion ratio was decreased by 0.20 and 0.27 (*p* < 0.05), respectively. In relation to flesh quality, the addition of Hyp + VC significantly increased the flesh contents of total collagen, heat-insoluble collagen, flavor-free amino acids, flesh hardness, muscle fiber density, and glutathione catalase activity (*p* < 0.05), as well as significantly reducing the cooking loss and muscle fiber diameter (*p* < 0.05). The EU group also showed a significantly higher flesh hardness, flavor-free amino acid content, muscle fiber density, and total antioxidant capacity, as well as a lower fiber diameter, than the control group (*p* < 0.05). In conclusion, the dietary supplementation of 14 g/kg coated Hyp (50%) + 2 g/kg L-ascorbyl-2-phosphate (38%) or 20 g/kg EU improved the growth performance and flesh quality of *L. vannamei*.

## Simple Summary

With the continuous expansion of Pacific white shrimp (*Litopenaeus vannamei*) culture in recent years, consumers are paying more attention to shrimp flesh quality. The study investigated the effects of dietary hydroxyproline (Hyp) + vitamin C (VC) and *Eucommia ulmoides* (EU) on the growth performance and flesh quality of Pacific white shrimp. Based on a practical diet with 200 g/kg fish meal inclusion (control diet), 14 g/kg coated Hyp (50%) + 2 g/kg L-ascorbyl-2-phosphate (38%) (Hyp + VC) or 20 g/kg EU was added to form three iso-nitrogenous diets. After 8 weeks of feeding, dietary Hyp + VC and EU significantly increased the weight gain, flesh hardness, myofibre density, and flavor-free amino acid content, as well as reducing the feed conversion ratio and flesh steaming loss of *L. vannamei.*

## 1. Introduction

Pacific white shrimp (*Litopenaeus vannamei)* is an important aquaculture shrimp in China and globally due to its high nutritional value and delicious flavors. With increasing shrimp aquaculture in recent years, consumers are becoming more conscious of the flesh quality. Flesh quality serves as a comprehensive index, which can be evaluated by sensory indicators, chemical composition, physical parameters, histological characteristics, and flavor characteristics. A good flesh quality can promote the purchasing power of consumers, thus ensuring the sustainable development of shrimp aquaculture.

*Eucommia ulmoides Oliv* (EU) is a traditional medicinal plant with multiple functions such as antioxidant properties, lowering blood pressure, enhancing immunity, and improving flesh quality [1]. The major active components in EU are flavonoids, phenolic acids, iridoids, and lignans [2]. Among them, flavonoids, selenium, vitamin E, and other antioxidants can strengthen the antioxidant capacity of cell biofilms and lipids to protect flesh quality [3]. As a herbal medicine additive, EU has been used in the diets of large yellow croaker (*Larimichthys crocea*) [3], Songpu silver crucian carp (*Carassius auratus gibelio var.* CAS V) [4], and red crayfish (*Cherax quadricarinatus*) [5] to improve their growth performance. In addition, dietary EU (20 g/kg) increased the flesh collagen content of *Litopenaeus vannamei* [1]. The dietary addition of 4 g/kg EU bark extract or 4 g/kg EU leaf extract increased the contents of calcium, total collagen, and heat-insoluble collagen in the muscle of grass carp (*Ctenopharyngodon idellus*) [6]. In rainbow trout (*Oncorhynchus mykiss*), the flesh quality, including its elasticity, myofiber fiber diameter, and myofiber fiber density, was also improved by the supplemental water extract of EU bark (40 g/kg) [7].

Collagen, as the main component of connective tissue, plays an important role in maintaining the stability and integrity of muscle tissue structure, which is also a crucial element affecting flesh quality [8]. Hydroxyproline (Hyp) is the characteristic amino acid in collagen. Studies have shown that adding Hyp to high plant protein feed promoted the growth and muscle collagen content of turbot (*Scophthalmus maximus* L.) [9]. The addition of Hyp to the diet also increased the collagen content in the swim bladder tissue of *Nibea diacanthus* juveniles [10]. In the formation of collagen, VC plays an important role as the cofactor of hydroxylase for the hydroxylation conversion of proline and lysine to Hyp and hydroxylysine [11]. Dietary VC (59.1 mg/kg) has been reported to reduce the flesh steaming loss and drip loss, as well as improving the growth performance, of *Trachinotus ovatus* [8]. The supplementation of 100 mg/kg or 200 mg/kg of VC to the diet also significantly increased the muscle collagen content of grass carp [12].

Previous studies have shown the improved growth performance and flesh quality of *L. vannamei* using dietary Hyp and VC, including muscle texture characteristics and water holding capacity [13], which indicated a possible synergistic effect between Hyp and VC. In terms of EU, some reports have been conducted on improving the meat quality of shrimp, but the evaluation indicators are relatively simple, focusing on muscle nutritional value and collagen content [1]. The effects of dietary EU on the flesh texture characteristics, histology, and flavor substances are still unclear. In addition, it is not known whether the effects of EU and Hyp + VC on the growth and flesh quality of shrimp are similar or not; both areas need further study. Therefore, in the current investigation, Hyp + VC or EU was added to the feed to compare the effects on the growth performance and flesh quality of Pacific white shrimp. The findings will provide a basis for improving the flesh quality of *L. vannamei*.

## 2. Materials and Methods

### 2.1. Ethical Statement

All the procedures for handling animals involved in this experiment were in accordance with the regulations of the Experimental Animal Ethics Committee and the Institutional Animal Care Committee of Shanghai Ocean University.

### 2.2. Experimental Design and Diets

A practical feed with a fish meal content of 200 g/kg was used as the control group (CON); then, 20 g/kg EU or 14 g/kg coated Hyp (50%) + 2 g/kg L-ascorbate-2-phosphate (VC content of 38%) (Hyp + VC) was added to form three experimental feeds. The supplemental levels of EU and Hyp + VC referred to the reports of Liu et al. [1] and Huang et al. [13]. When making feeds, the solid raw materials were crushed and sieved through a 60-mesh sieve, before being combined with oil and water. The mixture was extruded using a single screw extruder (LX-75 feed extruder, Hebei Longxiang Machinery) to form sinking pellets with a diameter of 1.2 mm (pelleting temperature of 85 ± 5 °C). After the granulation, the pellets were post-cooked in an oven at 95 °C for 20 min, and were then dried, sealed, and stored in plastic bags. The diet formula and proximate composition are presented in Table 1, and the amino acid composition is shown in Table 2.

EU was provided by Yinghuangshenrong Products Co., Ltd., Jieyang, China. L-ascorbic acid-2-phosphate was supplied by Sampo Biochemical Technology Co., Ltd. (Beijing, China) with a VC content of 38%. Hyp (with a purity of ≧98%) was provided by Yuanye Biological Co., Ltd., (Shanghai, China). When used, Hyp and starch (1:1) were mixed with water at ratio of 1:1, and were then gelatinized at 90 °C for 30 min, dried at 105 °C, and crushed to form a coated product.

### 2.3. Experimental Animals and Feeding Management

Pacific white shrimp larvae (7 days old) were purchased from a commercial farm in Qingpu, Shanghai, and temporary feeding was carried out at the Binhai Aquaculture Station of Shanghai Ocean University (Shanghai, China). During the temporary feeding period, the shrimps were fed with commercial feed containing 40% crude protein and 6% crude lipid for four weeks. Then, 600 healthy shrimps (initial weight 0.7 ± 0.1 g) were randomly allotted into 12 cages (1.0 m × 1.2 m × 1.0 m) in 2 cement pools (5.0 m × 3.0 m × 1.2 m). Each cage contained 50 shrimps. There were 3 treatments and 4 replicates per treatment (cage). The shrimp were fed four times a day (7:00, 12:00, 17:00, and 22:00). According to the feeding behavior, water temperature, and weather, the amount of feed intake was adjusted to ensure no feed residue in the 2h after feeding. Every 3–4 days, the water was exchanged about 1/3, and the feces waste was cleaned via siphoning every 7 days. During the culture period, the water quality, in terms of temperature, salinity, dissolved oxygen, pH, ammonia nitrogen, and nitrite, was 26–32 °C, 0.5‰–1.0‰, ≥5.2 mg/L, 7.6–8.7, ≤0.05 mg/L, and ≤0.2 mg/L, respectively. The breeding experiment lasted for 8 weeks.

### 2.4. Sample Collection

Before the feeding experiment, 20 shrimps were taken and stored at −20 °C for an analysis of their initial composition. After the feeding experiment, the shrimps were starved for 24 h. The final number and weight of shrimps in each cage were recorded; then, weight gain (WG, %), feed intake (FI), feed conversion ratio (FCR), and survival were calculated. Five shrimps were chosen from each cage to measure body length and body weight to calculate condition factor (CF). Lymphatic blood was taken from the pericardium, and was then placed at 4 °C for 12 h, centrifuged at 4000 r/min for 10 min, and the supernatant was placed at −80 °C for the determination of biochemical indicators. The hepatopancreas was weighed to calculate the hepatosomatic index (HSI). After the shell was peeled off, the muscle was weighed to calculate meat yield (MY). The muscle of the sixth abdominal segment was taken and placed in fixed liquid, followed by slicing, and the remaining muscles were stored at −80 °C for the determination of amino acids, biochemical indicators, and collagen content. Two shrimps from each cage were boiled (100 °C) for 3 min, and the lightness (L*), red-green (a*), and yellow-blue (b*) values of the body surface were measured on both sides of the second abdominal segment. After shelling, the muscle of the second abdominal segment was taken for texture determination, and the third and fourth segments were taken for the determination of shear force. Another two shrimps were taken from each cage to peel off the shell, and the muscle was divided into three parts for the determination of cooking loss and thawing loss.

### 2.5. Measurement Indicators and Methods

#### 2.5.1. Growth Performance, Body Indices, and Nutrient Retention

Growth performance, including WG, FI, FCR, and survival, were calculated as follows:

Weight gain (WG, %) = 100 × [(final weight (g) − initial weight (g))/initial weight (g)]

Feed intake (FI, g/shrimp) = total feed intake/[(initial number + final tail number)/2]

Survival (%) = 100 × (final shrimp number∕initial shrimp number)

FCR = feed intake (dry weight, g)/[final weight (g) − initial weight (g)]

Condition factor (CF, g/cm^3^), hepatopancreas somatic indices (HSI, %), and meat yield were calculated as follows:

CF (g/cm^3^) = (whole body weight (g)/body length (g)^3^) × 100

HSI (%) = (hepatopancreas weight (g)/whole body weight (g)) × 100

Meat yield (%) = (Total abdominal and tail muscle weight (g)/whole body weight (g)) × 100

These measurements referred to Yao et al., (2022) [14].

#### 2.5.2. Muscle Textural Properties, Water Holding Capacity, and Body Color

The whole shrimp was boiled for 3 min before being shelled, and the muscle at the second abdominal segment was used for texture analysis using a Universal TA Texture Analyzer (Shanghai Tengba Instrument Technology Co., Ltd., Shanghai, China). A 25 mm × 25 mm cylindrical probe was selected with the target mode of deformation, and the setting variable was 40% with a time of 2 s and a test speed of 1 mm/s. The muscles of the 3rd and 4th abdominal segments were placed vertically in the direction of the cutter, and the maximal shear force was recorded when they were crushed by the cutter.

Water holding capacity: The third abdominal segment of the shrimp was weighed and recorded as W_1_ after peeling. Then, the flesh was steamed or boiled for 5 min. After the flesh cooled to room temperature and the surface moisture was wiped off, the flesh was weighed as W_2_. Another block of flesh was placed at −20 °C for 24 h, and was then thawed at room temperature and weighed as W_2_ after the surface water was removed. The formulae for calculating steaming loss, cooking loss, and thawing loss are as follows.

Steaming (boiling, thawing) loss (%) = 100 × [(W_1_ (g) − W_2_ (g)/W_1_ (g)]

Body color: the whole shrimp was boiled for 3 min, then a colorimeter (WSC–S colorimeter, o/d light source, glossy, stability ΔY ≤ 0.6, (Shanghai Precision Scientific Instrument Co., Ltd., Shanghai, China) Physical Optics Instrument Factory, Shanghai, China) was used to determine the values of L*, a*, and b* of the body surface on both sides of the second abdominal segment [14].

#### 2.5.3. Proximate Composition of the Diets, Shrimp, Muscle, and Amino Acid Composition of Muscle

The AOAC standard method was used to determine the crude protein, crude ash, and moisture content of shrimp, muscle, and diet samples (AOAC, 2005). Moisture was determined by drying the sample to a constant weight in an oven at 105 °C. After the moisture was determined, the dried sample was crushed for subsequent determination. The sample was burned for six hours at 550 °C in a muffle furnace to estimate the crude ash content. An automatic Kjeltec nitrogen analyzer (Kjeltec 2300, Foss, Sweden) and the chloroform–methanol method were used to detected crude protein and crude lipid contents, respectively.

The Hyp content in the muscle was determined using the alkaline hydrolysis method with a detection kit produced by Nanjing Jiancheng Bioengineering Institute (Nanjing). The detection principle is that the oxide generated by Hyp and the oxidant reacts with dimethylaminobenzaldehyde to form a purplish-red substance. Based on the nitrogen-protein factor of 6.25 and the Hyp content of 12.5% in collagen, the collagen content can be calculated by multiplying the Hyp content by a factor of 8.

#### 2.5.4. Muscle Amino Acids and Free Amino Acids

Amino acid composition of muscle: In total, 20 mg of freeze-dried muscle sample was placed in an amperometric flask; then, 10 mL of 6M hydrochloric acid was added to hydrolyze the sample at 110 °C in a vacuum environment for 24 h. Hydrolysate (0.5 mL) was vacuum dried, diluted, and filtered, and the composition and contents of amino acids were determined using an automatic amino acid analyzer (S-433D, Sekam, Germany).

Free amino acids in muscle: In total, 0.3 g of muscle sample (wet sample) was homogenized with 9 mL 5% trichloroacetic acid (TCA) and ultrasonically shaken in ice water for 15 min, before being placed at 4 °C for 2 h and centrifuged at 4 °C and 10,000 r/min for 10 min. The supernatant was collected and the pH was adjusted to 2.0 ± 0.2 with 6 M NaOH. The water phase membrane was filtered with a 0.22 μm water phase membrane, and the composition and content of free amino acids were determined using an automatic amino acid analyzer (S-433D, Saikam, Germany).

#### 2.5.5. Serum and Flesh Biochemical Indices

Serum total protein (TP), glucose (GLU), cholesterol (CHO), triglycerides (TGs), superoxide dismutase (SOD), glutathione peroxidase (GSH-PX) activity, malondialdehyde (MDA), and total antioxidant capacity (T-AOC) were measured using a microplate reader (BioTcK Gen5). All the measurements referred to the description of Li et al. [15] using kits supplied by Nanjing Jiancheng Bioengineering Institute, China [15].

#### 2.5.6. Muscle Histology

Muscle samples were dehydrated, hyalinized, and embedded in paraffin, before being sliced, stained with hematoxylin–eosin (HE staining), and fixed with neutral resin. The morphological structure of the muscle was observed and photographed using an imaging microscope (Nikon 80i, Tokyo, Japan) [16]. The number of muscle fibers in the sections was recorded to calculate muscle fiber density.

### 2.6. Statistical Analysis

The mean ± standard deviation (SD) was used to display the data results. The SPSS 26.0 statistical analysis program was used to process and analyze the relevant data. All data were subjected to a one-way analysis of variance (ANOVA) test. Multiple comparisons between groups were performed using the Tukey multiple range test. Data were considered significantly different from the other if *p* < 0.05.

## 3. Results

### 3.1. Growth Performance and Physical Indices

As shown in Table 3, there was no significant difference in survival, feed intake, and condition factor among the three groups (*p* > 0.05). Compared with the control group, the addition of Hyp + VC and EU increased the weight gain by 13.9% and 18.4% (*p* < 0.05), and decreased FCR by 0.20 and 0.27 (*p* < 0.05). The addition of Hyp + VC or EU in feed significantly decreased the HSI and increased the meat yield (*p* < 0.05).

### 3.2. Muscle Texture, Shear Force, Water Holding Capacity, and Body Color

As shown in Table 4, there were no significant differences (*p* > 0.05) in springiness, chewiness, resilience, shear force, boiling loss, and thawing loss of muscle among the groups. Dietary Hyp + VC and EU significantly increased muscle hardness and decreased steaming loss (*p* < 0.05). In body color, the yellowness (b*) of the EU group was significantly higher than that of the control group (*p* < 0.05), and no significant difference in lightness (L*) and redness (a*) was observed among the three groups (*p* > 0.05).

### 3.3. Whole Shrimp, Muscle Composition, and Muscle Collagen Level

As shown in Table 5, there was no significant difference in whole body composition, muscle moisture, crude lipid, and heat-soluble collagen contents among all the groups (*p* > 0.05). The crude protein content of muscle in the Hyp + VC group and the EU group was significantly higher than that in the control group (*p* < 0.05), and the crude ash content of muscle in the EU group was significantly higher than that in the other two groups (*p* < 0.05). Compared with the control group, the total collagen and heat-insoluble collagen in the Hyp + VC group were significantly increased (*p* < 0.05). The total collagen and heat-insoluble collagen in the muscle of the EU group also showed an increasing trend (*p* > 0.05).

### 3.4. Flesh Amino Acid Content

As shown in Table 6, there was no significant difference (*p* > 0.05) in the flesh amino acids content among all groups. As shown in Table 7, a total of 17 free amino acids were detected in shrimp flesh, among which glycine had the highest level, followed by arginine and proline. Compared with the control group, the total free amino acid contents of the Hyp + VC and EU groups were increased significantly (*p* < 0.05). In addition, the EU group presented significantly higher levels of total free amino acids, glycine, and alanine (*p* < 0.05), as well as a lower leucine level than the control group (*p* < 0.05). The flavor amino acids and glycine levels in the Hyp + VC group were significantly lower than those in the control group (*p* < 0.05).

### 3.5. Serum and Flesh Chemical Analysis

As shown in Table 8, there was no significant difference in the hemolymph contents of total protein, triglyceride, and glucose among the groups (*p* > 0.05). Dietary EU significantly decreased the total cholesterol content in the serum of shrimp (*p* < 0.05).

No significant difference in superoxide dismutase activity and MDA content in flesh was detected in the three groups (*p* > 0.05). The total antioxidant capacity of the muscle in the EU group was significantly higher than that of the control group (*p* < 0.05). The Hyp + VC group showed significantly higher glutathione peroxidase activity in the muscle than the other two groups (*p* < 0.05).

### 3.6. Muscle Tissue Structure

**Figure 1:**
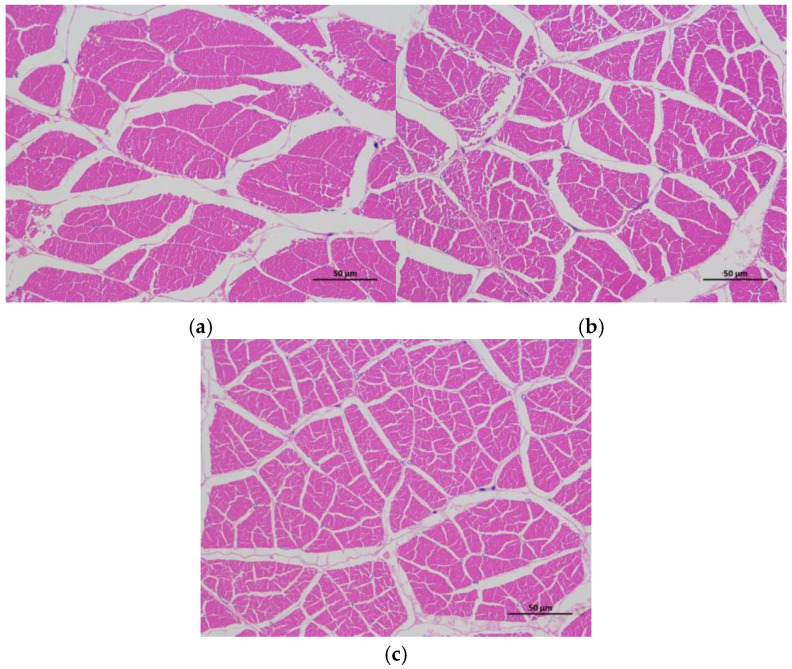
Muscle fiber structure of *L. vannamei* (H&E staining) fed with Hyp + VC and EU diets. (**a**), (**b**), and (**c**) represent the control, Hyp + VC, and EU groups, respectively.

In Figure 1 and Table 9, the myofiber diameter of the Hyp + VC group and the EU group was significantly smaller, and the myofiber density was significantly higher than that of the control group (*p* < 0.05).

## 4. Discussion

### 4.1. Effects of Hyp + VC and EU on the Growth Performance of L. vannamei

Hyp is a conditional essential amino acid for aquatic animals [17,18], which has the function of scavenging oxidants and regulating cell redox states. Studies have shown that the addition of 0.5%, 1%, and 1.5% proline to feeds containing 31% fishmeal and 15% fermented soybean meal significantly increased the feed intake, SGR, and WG of Mandarin fish (*Siniperca chuatsi*) [19]. When 50% and 60% of dietary fish meal was substituted with plant proteins, the supplementation of 0.6% Hyp significantly increased the specific growth rate of turbot [9]. In *Nibea coibor* juveniles, the dietary addition of 0.5% and 0.75% Hyp also significantly increased the WG [10].

Shrimps cannot synthesize VC in their bodies; thus, they need to obtain VC from their diets [20]. The growth performance of shrimps is also closely related to the amount of vitamin C in the feeds [21]. VC plays an important role in enhancing body resistance, promoting wound healing, crustacean growth, and protein metabolism [21,22]. In *Macrobrachium malcolmsonii* [23] and *L. vannamei* [24], it has been reported that dietary supplementation of VC significantly improved the survival and weight gain of shrimp.

Currently, the combined addition of Hyp + VC in aquatic feeds has been reported in *L. vannamei* by Huang et al. [13]. In that study, the addition of 14 g/kg Hyp + 2 g/kg VC to the diet increased the weight gain by 13.4% and reduced the FCR by 0.18 [13]. In the present study, the dietary supplementation of Hyp + VC also significantly increased the weight gain and meat yield, as well as decreasing the FCR (*p* < 0.05). Hydroxyproline is produced by the post-translational hydroxylation of proline in collagen via VC-dependent proline hydroxylases [17], which significantly affects the structural and physical properties of collagen [25]. Collagen is an important skeleton structural protein in animal cells, which can form high-strength insoluble fibers and play a supporting role in the body [26]. VC can enhance the antioxidant capacity of *L. vannamei*, thereby reducing the negative impact of stress. It is suggested that the synergistic effects of Hyp and VC may promote the growth of *L. vannamei*.

At present, EU has been applied in some aquatic feeds. The dietary addition of 2.0% EU significantly increased the WG and reduced the FCR of *L. vannamei* (*p* < 0.05) [1]. Li et al. [27] reported that the growth performance and survival of *L. vannamei* were improved by EU leaf extract (0.3 g/kg) supplementation. Tan et al. [28] found that a Chinese herbal medicine preparation with EU as the main component effectively improved the growth performance of *Megalobrama amblycephala*. In this experiment, the addition of 2% EU also significantly increased the weight gain and meat yield of *L. vannamei*, as well as reducing the FCR (*p* < 0.05). EU can promote the growth of farmed animals, which may be related to active components, such as chlorogenic acid and geniposide, in EU, thus improving the health, growth, and immunity [29].

### 4.2. Effects of Hyp + VC and EU on the Flesh Quality of L. vannamei

Shrimp body color is a visual characteristic of quality and freshness. Astaxanthin is the main pigment reflecting the body color of shrimp. Aquatic animals cannot synthesize astaxanthin de novo, so they need to obtain astaxanthin or an astaxanthin precursor from their diet [30,31,32]. In this experiment, no significant difference in body surface was observed between the Hyp+VC group and the control group (*p* > 0.05). This suggests that Hyp and VC may not affect the absorption and deposition of astaxanthin. However, the b* (yellowness) of the shrimp in the EU group was significantly higher than that in the control group (*p* < 0.05), indicating that EU might improve the surface color of shrimp; however, this needs further study.

The water holding capacity is an important quality trait affecting the color, flavor, juiciness, and tenderness of flesh. A lower cooking loss indicates that the nutrients and flavor can be well maintained in the flesh. In this experiment, the steaming loss of the Hyp + VC group and the EU group was significantly lower than that of the control group (*p* < 0.05), which indicated that the water holding capacity of the muscle was improved. The improved water holding capacity may be connected with the reduction in oxidative damage and the improvement in muscle integrity [33] because VC and EU have the function of reducing tissue oxidative damage and antioxidation.

The hardness, elasticity, resilience, and other parameters can be used to describe the texture properties of shrimp meat, and shear force is also considered as a biomarker of hardness [34]. These characteristics are closely related to the moisture, lipid, and collagen contents of the muscle. Collagen is the main component in skeletal muscle connective tissue and the main factor affecting intrinsic muscle hardness. Hyp is essential for collagen synthesis, which determines the texture of the meat; therefore, the amount of Hyp also affects the quality of the muscle [35]. In this experiment, the addition of Hyp + VC or EU significantly increased the muscle hardness (*p* < 0.05), and the total collagen and heat-insoluble collagen contents of the Hyp + VC group were also significantly higher than those of the control group (*p* < 0.05). The dietary addition of 200 or 800mg/kg of vitamin C significantly increased the muscle hardness of juvenile hybrid sturgeon (*Acipenser schrenckii* × *A. baeri*) [36], while the addition of hydroxyproline to the diet of triploid crucian carp (*Carassius auratus triploid*) increased the content of collagen in the muscle [37]. Similar results were also reported in relation to EU for grass carp [38] and *L. vannamei* [1]. The combined addition of Hyp + VC improved the flesh quality of *L. vannamei* in the current study. Hyp provides the raw material for the synthesis of collagen, and VC is involved in the formation of collagen as a coenzyme of proline hydroxylase and lysine hydroxylase. Hyp and VC may have synergistic effects in the synthesis of collagen. EU also has the role of promoting collagen synthesis, which may be related to its active ingredients such as chlorogenic acid, geniposidic acid, and aucubin [39]. Previous studies have shown that the muscle collagen content of grass carp was increased by the dietary addition of 400 mg/kg chlorogenic acid [6] or 600–1000 mg/kg geniposidic acid [39].

Myofibrils are the basic unit of muscle, and hypertrophy and hyperplasia are the two processes associated with myofibrillar growth [40,41]. Generally, the high density of myofibers and the small diameter of myofibers means a tight and dense muscle [42]. An increase in myofiber density and a decrease in myofiber diameter are responsible for an increase in muscle masticatory force, leading to an improvement in muscle quality [35]. Myofiber density has been reported to be positively correlated with collagen and Hyp content [43], whereas VC positively modulates skeletal muscle growth through antioxidant and anti-inflammatory effects [44]. Our previous study has shown that the addition of 14 g/kg Hyp + 2 g/kg VC significantly increased the muscle fiber density of *L. vannamei* [13]. EU contains chlorogenic acid, geniposide, geniposidic acid, and other active ingredients, which may be the main factors causing the increase in myofiber density [7]. In grass carp, the dietary addition of chlorogenic acid, geniposide, and geniposidic acid significantly increased the myofiber density [39]. The myofiber density of rainbow trout was also increased by EU bark water extract [7]. In addition, EU bark extract, EU leaf extract, chlorogenic acid, quercetin, and aucubin have been found to promote the intramuscular fibroblasts’ proliferation of grass carp [6]. In this study, the myofiber density of shrimp was also significantly increased by the supplemental Hyp + VC and EU, indicating that Hyp + VC and EU may promote the proliferation of myofibers.

The formation of flesh quality is largely related to the oxidation level of the muscle. The main antioxidant substances in the muscle include CAT, SOD, and other antioxidant enzymes. The total T-AOC reflects the comprehensive antioxidant potential of an organism [45]. GSH-PX and SOD are the main antioxidant enzymes in animals, and their activities directly affect the content of MDA [39]. Antioxidant enzymes (CAT, SOD, etc.) in the muscle can effectively inhibit the production and transfer of free radicals, reduce the oxidation degree, and prevent the decrease in nutritional value, sensory quality, and tenderness of the muscle [46]. In this experiment, the EU group present significantly higher muscle T-AOC than the control group (*p* < 0.05). This potentially suggests that chlorogenic acid, flavonoid glycosides, and lignans in EU exert antioxidant capacity, scavenge free radicals, and protect the normal cell metabolism of the body [29].

Flavor amino acids are the important material basis for the flavor presentation of aquatic products. The deliciousness of aquatic products is mainly determined by the composition and content of free-flavor amino acids, including Asp, Glu, Arg, Ala, and Gly. Glycine is the most important amino acid for collagen synthesis [47]. In this study, the addition of Hyp + VC increased the free Gly content in muscle in agreement with the reports on Yellow River carp (*Cyprinus carpio haematopterus*); Song et al. [48] indicated that the endogenous synthesis of Gly from Hyp is physiologically and nutritionally important, and the effect of dietary EU on Gly may be related to its active ingredients’ ability to promote collagen synthesis [39]. Meanwhile, the amount of free-flavor amino acids was significantly increased by the dietary supplementation of EU. Previous studies showed that the addition of 4% EU leaf powder to feed significantly increased the amount of flavor amino acids, essential amino acids, and total amino acids in the muscle of common carp (*Cyprinus carpio*) [49]; similar results have also been reported in grass carp [39] and black carp (*Mylopharyngodon piceus*) [3]. EU may stimulate the synthesis of proteins in the muscle, resulting in an increase in amino acids.

## 5. Conclusions

In the present study, the addition of 14 g/kg coated Hyp (50%) + 2 g/kg L-ascorbyl-2-phosphate (VC 38%) or 20 g/kg EU in feed promoted the growth and flesh quality of *L. vannamei* by increasing the weight gain, flesh hardness, myofiber density, and free-flavor amino acid content, as well as reducing the FCR and flesh steaming loss.

## Figures and Tables

**Table 1:** Formulation and proximate composition of diets (air-dry basis, g/kg).

Ingredients	CON	Hyp + VC	EU
Fish meal	200.0	200.0	200.0
Flour	270.0	270.0	240.0
Soybean meal	230.0	214.0	240.0
Soybean protein concentrate	100.0	100.0	100.0
Corn protein powder	50.0	50.0	50.0
Peanut meal	40.0	40.0	40.0
Squid visceral meal	50.0	50.0	50.0
Soy lecithin	15.0	15.0	15.0
Fish oil	15.0	15.0	15.0
Vitamin and mineral premix ^1^	25.0	25.0	25.0
Coated hydroxyproline (50%)	0.0	14.0	0.0
L-ascorbic acid-2-phosphate (38%)	0.0	2.0	0.0
*Eucommia ulmoides*	0.0	0.0	20.0
Total	1000.0	1000.0	1000.0
Proximate composition (g/kg)			
Crude protein	398.8	393.9	401.7
Crude lipid	62.9	60.5	63.4
Crude ash	83.3	86.8	90.4
Moisture	74.6	78.2	75.5

^1^ One kilogram of premix contains the following: Vit A: 400,000 IU; Vit D3: 180,000 IU; Vit E: 3 g; Vit K: 1 g; Vit B1: 0.5 g; Vit B2: 1.5 g; Vit B6: 0.8 g; Vit C: 16 g; D-biotin: 8 mg; folic acid: 0.24 g; niacinamide: 4 g; d-pantothenic acid: 2.5 g; inositol: 15 g; K: 42.9 g; Mg: 6 g; Fe: 0.5–72 g; Cu: 1.5–5 g; Zn: 5–12 g; Mn: 1.5–15 g; Co: 114–200 mg; Se: 1.8–50 mg; I: 30–100 mg; moisture ≤ 10%.

**Table 2:** Amino acid composition of diets (dry-matter basis, g/kg).

Items	CON	Hyp + VC	EU
Essential amino acids (EAAs)			
Threonine	16.0	15.8	16.3
Methionine	5.9	6.2	6.3
Valine	20.3	20.5	20.6
Isoleucine	16.0	16.1	16.4
Leucine	30.9	31.5	31.8
Phenylalanine	21.3	21.7	20.6
Histidine	12.3	12.3	12.5
Lysine	23.9	23.8	24.1
Arginine	25.4	26.2	25.3
Non-essential amino acids (NEAAs)			
Aspartic acid	37.8	37.2	38.5
Serine	17.7	17.4	17.4
Glutamic acid	75.6	75.6	78.7
Glycine	18.5	18.5	18.6
Alanine	18.3	18.3	18.5
Cysteine	3.9	5.4	5.6
Tyrosine	8.0	12.0	10.1
Proline	20.6	19.4	19.3
Total amino acids (TAAs)	372.4	377.9	380.6

**Table 3:** Dietary effects of Hyp + VC and EU on the growth performance and body indices of *L. vannamei*.

Items	CON	Hyp + VC	EU
IBW (g)	0.70 ± 0.04	0.70 ± 0.02	0.70 ± 0.05
FBW (g)	8.06 ± 0.19 ^b^	9.31 ± 0.01 ^a^	9.82 ± 0.22 ^a^
WG (%)	1060.9 ± 25.5 ^b^	1210.0 ± 36.2 ^a^	1257.9 ± 80.1 ^a^
Survival (%)	76.0 ± 5.29	80.0 ± 5.29	80.6 ± 5.03
FI (g/shrimp)	14.77 ± 1.05	15.14 ± 0.56	14.72 ± 0.67
FCR	1.89 ± 0.10 ^a^	1.69 ± 0.04 ^b^	1.62 ± 0.08 ^b^
CF (g/cm^3^)	0.80 ± 0.07	0.78 ± 0.06	0.72 ± 0.01
HSI (%)	3.99 ± 0.59 ^a^	2.78 ± 0.19 ^b^	2.74 ± 0.18 ^b^
MY (%)	47.43 ± 0.29 ^b^	55.10 ± 0.61 ^a^	54.27 ± 2.24 ^a^

Data with different letters in the same row indicate significant differences (*p* < 0.05), the same applies to the following tables. IBW: initial body weight; FBW: final body weight; FI: feed intake; WG: weight gain; FCR: feed conversion ratio; HSI: hepatopancreas–somatic index; CF: condition factor; MY: meat yield.

**Table 4:** Dietary effects of Hyp + VC and EU on the muscle texture, shear force, water holding capacity, and body surface color of *L. vannamei*.

Items	CON	Hyp + VC	EU
Hardness/gf	897.2 ± 9.9 ^b^	1168.2 ± 36.7 ^a^	1088.2 ± 87.6 ^a^
Springiness/gf	0.58 ± 0.01	0.57 ± 0.2	0.58 ± 0.02
Chewiness/gf	349.1 ± 2.9	427.5 ± 27.9	414.6 ± 26.6
Cohesiveness/gf	0.67 ± 0.01	0.64 ± 0.01	0.65 ± 0.02
Resilience	0.62 ± 0.02	0.61 ± 0.03	0.61 ± 0.01
Shear force/gf	884.1 ± 80.2	902.1 ± 80.8	888.2 ± 62.5
Steaming loss/%	26.42 ± 0.27 ^a^	20.68 ± 1.88 ^b^	20.18 ± 0.01 ^b^
Boiling loss/%	24.94 ± 1.13	24.06 ± 1.18	22.15 ± 3.41
Thawing loss/%	2.56 ± 0.26	2.42 ± 0.07	2.23 ± 0.35
Body color			
Lightness L*	51.77 ± 0.92	50.17 ± 0.67	51.53 ± 1.05
Redness a*	19.53 ± 0.97	22.16 ± 0.35	22.13 ± 1.36
Yellowness b*	28.26 ± 1.51 ^b^	31.10 ± 1.04 ^ab^	32.50 ± 1.65 ^a^

In the same row, values with different small letter superscripts mean significant difference (*p* < 0.05).

**Table 5:** Dietary effects of Hyp + VC and EU on the whole body and flesh composition of *L. vannamei* (wet weigh basis, g/kg).

Items	CON	Hyp + VC	EU
Whole body			
Moisture	750.29 ± 16.21	750.73 ± 8.62	751.31 ± 11.30
Crude protein	170.71 ± 11.81	182.56 ± 3.10	183.15 ± 0.80
Crude lipid	26.22 ± 3.88	25.84 ± 10.16	24.62 ± 4.63
Crude ash	29.86 ± 1.76	31.33 ± 1.21	31.47 ± 0.36
Flesh			
Moisture	768.60 ± 0.21	767.30 ± 5.50	766.10 ± 0.22
Crude protein	178.8 ± 8.20 ^b^	203.9 ± 4.71 ^a^	203.5 ± 8.60 ^a^
Crude lipid	13.80 ± 0.60	16.40 ± 0.61	12.70 ± 0.40
Crude ash	12.00 ± 0.50 ^b^	11.10 ± 0.21 ^b^	13.60 ± 0.11 ^a^
Total collagen	2.16 ± 0.11 ^b^	3.12 ± 0.11 ^a^	2.48 ± 0.12 ^b^
HS collagen	0.55 ± 0.08	0.49 ± 0.16	0.50 ± 0.04
HIS collagen	1.61 ± 0.54 ^b^	2.63 ± 0.05 ^a^	1.98 ± 0.07 ^ab^

HS collagen: heat-soluble collagen; HIS collagen: heat-insoluble collagen. In the same row, values with different small letter superscripts mean significant difference (*p* < 0.05).

**Table 6:** Dietary effects of Hyp + VC and EU on the amino acid composition in the flesh of *L. vannamei* (dry matter basis, g/kg).

Items	CON	Hyp + VC	EU
Essential amino acids (EAAs)			
Threonine	26.41 ± 0.31	32.65 ± 0.26	29.14 ± 0.34
Phenylalanine	32.64 ± 0.23	34.67 ± 0.24	36.15 ± 0.02
Lysine	78.28 ± 0.31	72.12 ± 0.27	80.24 ± 0.13
Histidine	14.27 ± 0.23	17.41 ± 0.19	17.56 ± 0.26
Arginine	77.10 ± 0.56	83.25 ± 0.73	84.42 ± 0.78
Valine	34.81 ± 0.13	36.41 ± 0.45	37.85 ± 0.14
Methionine	13.52 ± 0.10	16.33 ± 0.16	13.82 ± 0.11
Isoleucine	33.16 ± 0.43	36.72 ± 0.25	34.45 ± 0.29
Leucine	64.13 ± 0.13	64.75 ± 0.45	64.71 ± 0.57
Non-essential amino acids (NEAAs)			
Tryptophan	25.42 ± 0.02	27.01 ± 0.11	27.12 ± 0.78
Aspartic acid	86.64 ± 0.27	88.82 ± 0.60	80.67 ± 0.39
Proline	44.36 ± 0.41	44.35 ± 0.31	41.43 ± 0.45
Serine	15.62 ± 0.12	16.62 ± 0.37	17.17 ± 0.55
Glutamic acid	155.21 ± 0.19	155.32 ± 0.61	162.02 ± 0.11
Glycine	78.53 ± 0.19	76.53 ± 0.44	79.94 ± 0.10
Alanine	58.24 ± 0.10	51.91 ± 0.36	58.12 ± 0.15
Cysteine	6.20 ± 0.04	7.64 ± 0.18	5.11 ± 0.02
TAAs	844.54 ± 6.76	862.51 ± 1.91	869.92 ± 4.34

**Table 7:** Dietary effects of Hyp + VC and EU on the free amino acid composition in the flesh of *L. vannamei* (fresh weight, mg/100 g).

Items	CON	Hyp + VC	EU
Aspartic acid	13.07 ± 0.87	13.95 ± 0.86	12.40 ± 0.02
Threonine	90.94 ± 9.62	93.55 ± 5.50	97.86 ± 12.11
Serine	13.07 ± 0.97	13.43 ± 0.77	14.02 ± 1.32
Glutamic acid	44.45 ± 1.35	48.42 ± 2.74	52.53 ± 3.90
Glycine	939.05 ± 24.42 ^c^	1017.35 ± 3.51 ^b^	1217.71 ± 4.91 ^a^
Alanine	160.12 ± 5.30 ^b^	174.67 ± 6.41 ^ab^	180.96 ± 7.32 ^a^
Cysteine	3.72 ± 0.26	4.18 ± 0.25	4.23 ± 0.31
Valine	14.28 ± 1.74	13.69 ± 1.18	16.17 ± 0.48
Methionine	5.61 ± 0.40	5.66 ± 0.34	5.46 ± 0.23
Isoleucine	7.41 ± 0.82	7.86 ± 0.98	7.41 ± 0.84
Leucine	15.81 ± 0.74 ^a^	14.71 ± 0.28 ^ab^	11.51 ± 0.43 ^b^
Tryptophan	14.43 ± 1.46	14.34 ± 0.87	15.34 ± 1.13
Phenylalanine	12.97 ± 0.87	11.76 ± 0.88	10.18 ± 1.08
Lysine	32.38 ± 2.19	31.90 ± 0.18	33.83 ± 1.17
Histidine	14.61 ± 0.34	14.85 ± 1.48	15.45 ± 0.82
Arginine	612.37 ± 15.28	636.45 ± 8.70	649.53 ± 19.32
Proline	490.54 ± 10.93	556.02 ± 22.26	495.13 ± 4.43
DAAs	1184.09 ± 16.29 ^c^	1280.51 ± 8.32 ^b^	1489.27 ± 0.19 ^a^
TAAs	2484.83 ± 22.14 ^c^	2672.82 ± 41.64 ^b^	2839.88 ± 1.87 ^a^

DAAs: flavor amino acids (Asp, Glu, Gly, and Ala); TAAs: total amino acids. Data with different letters in the same row indicate significant differences (*p* < 0.05).

**Table 8:** Dietary effects of Hyp + VC and EU on the hemolymph and muscle biochemical indices of *L. vannamei*.

Items	CON	Hyp + VC	EU
Hemolymph			
TP (g/L)	62.72 ± 0.12	63.24 ± 1.54	63.54 ± 3.24
TG (mmol/L)	1.38 ± 0.21	1.09 ± 0.51	1.12 ± 0.32
T-CHO (mmol/L)	4.24 ± 0.28 ^a^	3.33 ± 0.43 ^ab^	2.65 ± 0.51 ^b^
GLU (mmol/L)	1.58 ± 0.24	1.57 ± 0.06	1.54 ± 0.19
Flesh			
SOD (U/mL)	169.93 ± 24.12	250.76 ± 12.67	200.66 ± 23.63
T-AOC (mg prot/mL)	8.13 ± 0.74 ^b^	9.79 ± 0.41 ^ab^	9.99 ± 0.31 ^a^
GSH-PX (mg prot/μmol)	125.29 ± 10.98 ^b^	150.37 ± 3.81 ^a^	124.50 ± 1.55 ^b^
MDA (nmol/mL)	1.89 ± 0.81	1.74 ± 0.55	2.09 ± 0.27

TP: total protein; TG: triglyceride; T-CHO: total cholesterol; GLU: glucose; MDA: malondialdehyde; SOD: superoxide dismutase; T-AOC: total antioxidant capacity; GSH-PX: glutathione peroxidase. Data with different letters in the same row indicate significant differences (*p* < 0.05).

**Table 9:** Dietary effects of Hyp + VC and EU on the myofiber density and diameter of *L. vannamei*.

Items	CON	Hyp + VC	EU
Myofiber diameter (μm)	172.9 ± 1.7 ^a^	120.7 ± 7.4 ^b^	130.9 ± 8.3 ^b^
Myofibre density (cell/mm^2^)	133.2 ± 1.2 ^b^	173.8 ± 3.6 ^a^	162.1 ± 7.8 ^a^

In the same row, values with different small letter superscripts mean significant difference (*p* < 0.05).

## Data Availability

Data are contained within the article.
